# Leading consultants in the emergency departments are more in favour of a specialty in emergency medicine than their collaborating colleagues

**DOI:** 10.1186/1757-7241-21-S2-A33

**Published:** 2013-09-09

**Authors:** Julie Mackenhauer, Nina Bjerre Andersen, Hans Kirkegaard

**Affiliations:** 1 Research Center for Emergency Medicine, Aarhus University Hospital, Denmark; 2 Centre for Medical Education, Aarhus University, Denmark; 3 Akutafdelingen, Sygehus Vendsyssel, Denmark

## Background

In 2007 the Danish Health and Medicines Authority decided to reorganize the acute care area, establishing common emergency departments (ED). As emergency medicine (EM) is not an independent specialty in Denmark, EDs are staffed by consultants from other specialties.

The aim of this study was to examine leading consultants’ - both from the ED and collaborating departments - knowledge of the EM discipline description (fagområdet), and their opinion on its contribution to the acute area, as well as potential benefits of an independent specialty.

## Methods

An electronic questionnaire was sent to leading consultants from the ED (September 2012) and collaborating departments (January 2013); ICU, surgery, orthopedics, internal medicine, cardiology, clinical biochemistry and radiology. Answers were reported on a 5-point Likert-scale(from Highly disagree-Highly agree) or yes/no.

## Results

101 of 137 collaborating leaders (74%), and 21 ED leaders (100%) replied.

95% (n=20) of ED leaders and 50% (n=49) of collaborating leaders have knowledge of the EM discipline description.

Of these 23% (n=16) highly agree and 30% (n=21) agree that a specialty in EM will have a more positive impact on the cooperation between EDs and collaborating specialties in comparison with the discipline. 17% (n=12) disagree and 9% (n=6) highly disagree, 20% (n=14) answered neither/nor. Comparing Likert-scale means, ED leaders had a significantly higher level of agreement (p=0,0470, Wilcoxon 2-sample test).

Answering if EM should be an independent specialty in Denmark, orthopedics or leaders from Region Sjælland stood out with median Likert-scores of 4.5 [3.75;5](IQR) and 4 [3;5](IQR) respectively, in contrast to leaders from internal medicine or Region Syddanmark, who had median Likert-scores of 2 [1.75;4.25](IQR) and 2 [2;3.75](IQR) respectively. ED leaders had a significantly higher level of agreement compared to collaborating leaders in general with 5 [3;5](IQR) vs. 3 [2;4](IQR)(p=0,0149, wilcoxon 2-sample test).

## Conclusion

Only 50% of collaborating leaders cooperating with the EDs know about the discipline description. Of leaders aware of the discipline description, 53% agree or highly agree an independent specialty, more than the discipline, will have positive impact on the cooperation between the ED and the collaborating department. Supporting an independent specialty varies with the leaders affiliation to specialty and region.

